# A *substrate* for modular, extensible data-visualization

**DOI:** 10.1186/s41044-019-0043-6

**Published:** 2020-02-10

**Authors:** Jordan K. Matelsky, Joseph Downs, Hannah P. Cowley, Brock Wester, William Gray-Roncal

**Affiliations:** The Johns Hopkins University Applied Physics Laboratory, Laurel, MD, USA

**Keywords:** Visualization, Data science, Big data, Graph visualization, WebGL, Browser, Jupyter, Python, JavaScript

## Abstract

**Background:**

As the scope of scientific questions increase and datasets grow larger, the visualization of relevant information correspondingly becomes more difficult and complex. Sharing visualizations amongst collaborators and with the public can be especially onerous, as it is challenging to reconcile software dependencies, data formats, and specific user needs in an easily accessible package.

**Results:**

We present substrate, a data-visualization framework designed to simplify communication and code reuse across diverse research teams. Our platform provides a simple, powerful, browser-based interface for scientists to rapidly build effective three-dimensional scenes and visualizations. We aim to reduce the limitations of existing systems, which commonly prescribe a limited set of high-level components, that are rarely optimized for arbitrarily large data visualization or for custom data types.

**Conclusions:**

To further engage the broader scientific community and enable seamless integration with existing scientific workflows, we also present pytri, a Python library that bridges the use of substrate with the ubiquitous scientific computing platform, *Jupyter.* Our intention is to lower the activation energy required to transition between exploratory data analysis, data visualization, and publication-quality interactive scenes.

## Background

Using modern web-based visualization frameworks [1, 2] makes it easy to generate beautiful, interactive, and informative visualizations of scientific data. These renderings simplify the processes of exploring data and sharing insights with the community. In many domains, this has become a key step in the research and discovery pipeline [3].

One challenge with these technologies is the difficulty of adapting prior visualization work to a new use-case. These tools are often built to be single-purpose rather than interoperable. Therefore it can be difficult or even impossible to combine aspects of disparate visualization scenes, even when the visualizations use the same technologies or frameworks. This challenge leads to software duplication instead of reuse, and complicates the portability of these products between research efforts. Often, the developers of modern visualization systems have chosen to either enjoy wide adoption at the expense of domain-specific tooling (e.g., plotly and matplotlib), or have focused on scientific subdomains at the expense of extensibility (e.g., common GIS or biological rendering software such as neuroglancer [4] and FreeSurfer [5]). As a result, combining visuals from more than one analysis or modality often requires significant engineering effort [6].

Scientists and software developers have produced several frameworks designed to remedy these challenges [3, 7, 8] and we leverage architectural ideas from some of these frameworks in our solution, called substrate. We follow a compositional model similar in spirit to component-based engines such as React or Vue [4, 8–10], wherein a visualization is comprised of many individual, independent components. Furthermore, we have developed pytri, a Python module that enables Python developers to access and interact with WebGL-based substrate from inside a Jupyter environment without requiring prior JavaScript or WebGL knowledge. This ecosystem includes integrated Jupyter notebook capabilities found in systems such as Mayavi or Neuroglancer [4, 7] alongside generalized visualization capabilities found in systems such as Plotly or Matplotlib [11, 12].

Unlike many existing Jupyter visualization packages, pytri visualizations are fully customizable, even by the end user. Users are not constrained by the limits of prepackaged visualization data structures or plot types, and can combine prebuilt components alongside custom, purpose-built visualization components. Users directly interact with their underlying data as usual, while these tools bring visualization capabilities fully into the data analytics platform.

Our tools are designed to enable the visualization of large-scale data or custom datatypes, coregister multimodal data, and simplify the process of sharing or reproducing analyses — all without disrupting the data science process.

We have designed our approach to be usable with minimal technical knowledge: A user can enter this ecosystem with only a basic knowledge of JavaScript or Python, though power-users and proficient software developers will find these libraries easy to manipulate and extend to suit their needs.

We first describe the software design of substrate, and then introduce pytri in order to render substrate scenes from common Python data libraries such as numpy [13], networkx [14], or pandas [15]. Finally, we share example use-cases in which the interoperability provided by substrate can reduce the engineering overhead of a new visualization project. Code and tutorials are available online at the links in the *Availability of Data and Material* section.

### Implementation

To fully separate the responsibilities of substrate, our scene engine, and pytri, our Python integration library, our software architecture mandates that all rendering and Document Object Model (DOM) manipulation is handled by substrate and all Python manipulations and datatype translation take place in pytri.

### substrate

**Fig. 1:**
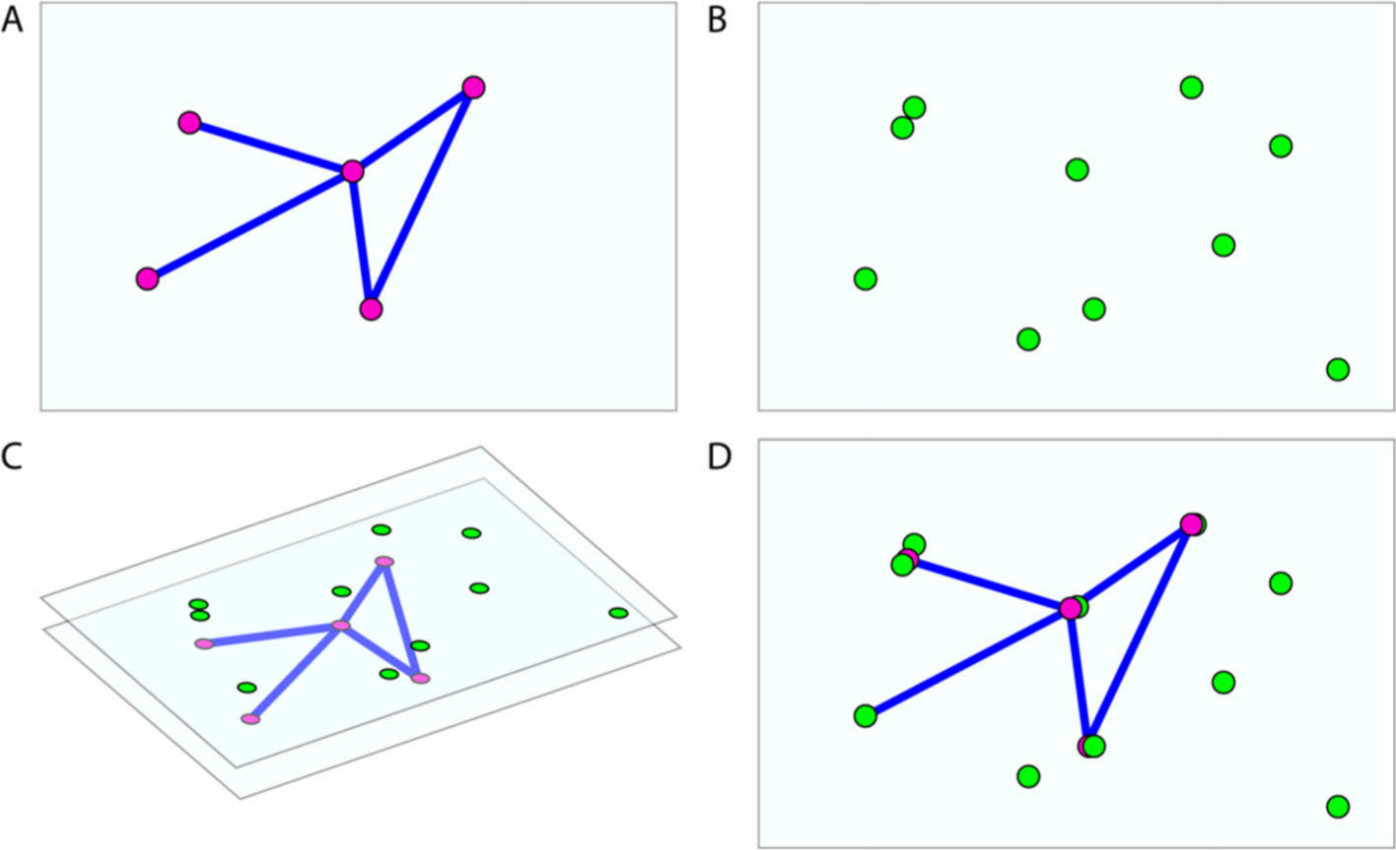
A GraphLayer and a ScatterLayer, embedded in the same 3D space. substrate overlays the two and controls the two independently. **a** One layer renders a graph object. **b** A second layer renders a scatter of points in 3D space. **c** The layers are composited by the substrate Visualizer into a single image. **d** The rendered scene, viewed from the substrate camera in finished form

substrate is a JavaScript library that exposes a simple but powerful developer-facing application program interface (API). This abstraction enables otherwise disparate visualization projects to share resources and logic easily. The substrate JavaScript library is intended to simplify component-reusability of commonly used data-visualization structures such as scatter plots or 3D-embedded graph visualizations. Each atomic visualization component implements a common Layer API. Each Layer in a scene handles its own data management and interactivity in isolation, while a parent Visualizer object manages a list of these Layers in much the same way as a React application re-renders its components as needed when its internal state changes [9]. For example, a ScatterLayer implements Layer by accepting an array of [x, *y,* z]-tuples, and it will render these data when initialized. In the same scene, a GraphLayer might render a 3D graph embedding, as shown in Fig. 1. These layers exist in the same 3D coordinate system, but are managed independently so changes to one does not require a re-render of the other components in the scene. Comprehensive Layer API documentation is available online.

We formalize a universal Layer interface that accommodates common visualization tasks. Layers must include:

1. requestInit (function) is called before the visualization starts: This function generally includes instructions to provision objects in a 3D scene, or to request data from a remote source with a long round-trip;
2. requestRender (function) runs on every frame. In static, non-animated Layers, this function may be empty or remain unimplemented in order to conserve compute power;
3. children (array attribute) lists all objects in a scene associated with a particular Layer. When a Layer is removed from the visualization, all objects in this list are cleaned up and garbage-collected by substrate internally.

This simple interface enables many different visualization objects or groups of objects to coexist in a scene without interfering with one another. Such namespacing conflicts are a common pitfall when combining conventional, separately-developed assets into a single scene using WebGL wrapper frameworks.

In order to improve the accessibility of our codebase to new developers and data scientists, we use three.js as a convenience to wrap WebGL. Despite the prevalence of three.js in our current codebase, substrate aims to be framework-agnostic. Authors of new Layers may choose to write WebGL directly, or use another wrapper or framework. substrate will support these Layers provided they subscribe to the substrate.Layer and substrate.Visualizer interfaces. In this way, substrate works in a similar extensible fashion to the Uber deck.gl project, though deck.gl re-implements full scene rendering from the ground up, whereas substrate uses the three.js industry standard [2].

**Fig. 2:**
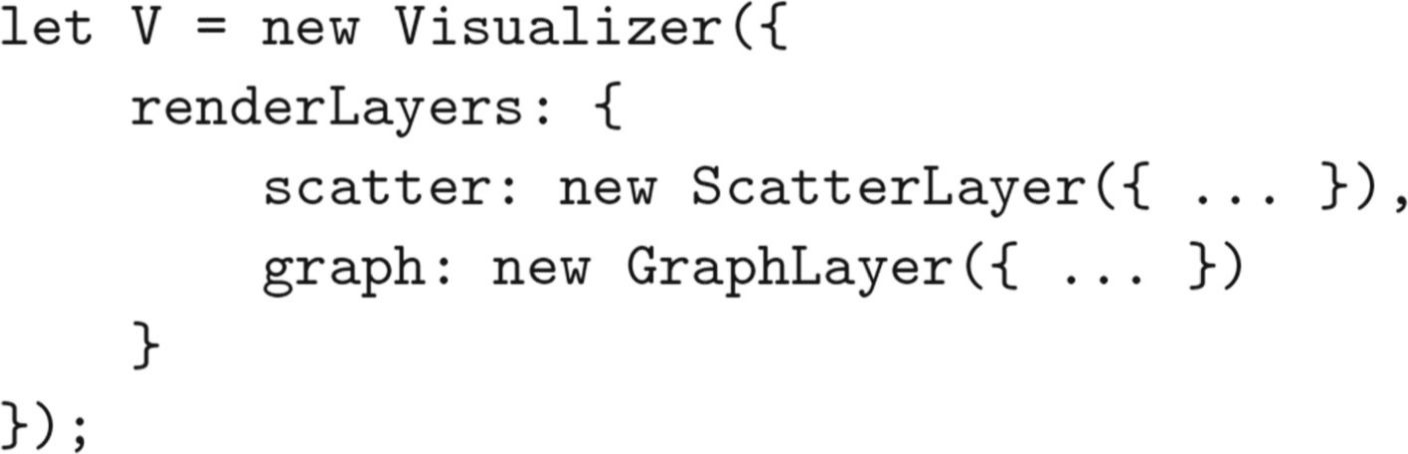
A sample Visualizer, with two Layers. One renders a 3D scatter-plot, and the other renders nodes and edges of an undirected graph

The compositional property of Layers can be expressed in code using the syntax shown in Fig. 2. Here we show a simple Visualizer containing two Layers; this short snippet can run a complete visualization without any extra configuration.

**Fig. 3:**
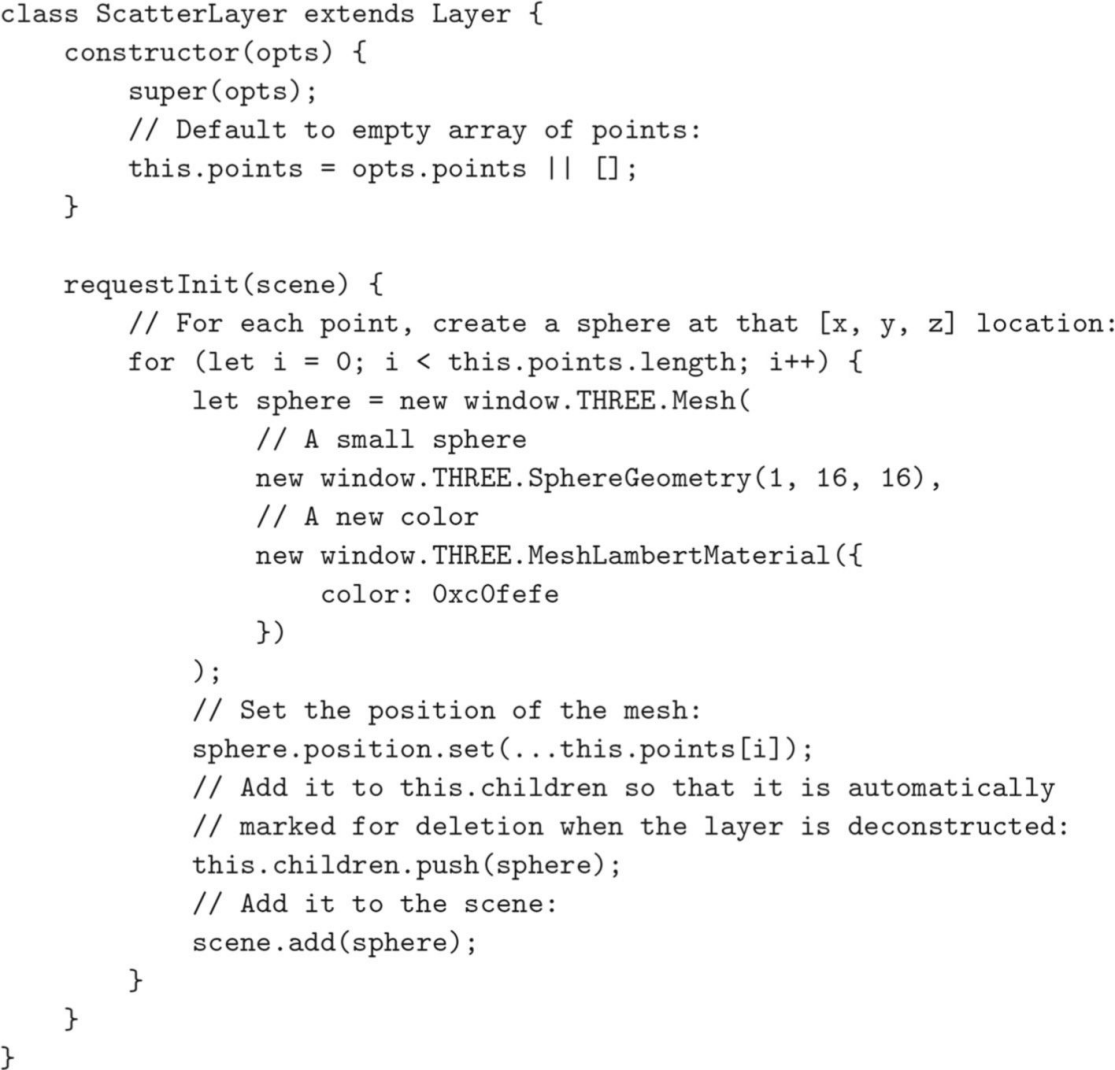
A sample implementation of a Layer that generates a point-cloud from the data provided in the constructor. This *exact implementation* can be dropped into any substrate visualization without modification. This code, and other Layer examples, are available online

One common use of separate Layers is to place objects of interest — such as a mesh — in one Layer, and place lighting, axes, or other environmental factors in another. This enables a researcher to share their core data, such as the 3D mesh, with other researchers, without including extraneous features such as light sources or grids and axes. (This simultaneously enables an artist to reuse their lighting layout across projects.) In Fig. 3, we illustrate a sample Layer implementation that can be ported to any substrate visualization. Our add-and-remove-layer demo provides an example in which a MeshLayer is added or removed, without affecting other objects in the scene.

Layers written for one visualization or application may be repurposed or reused in another scene without additional developer effort. This means that for most visualization use-cases, such as graph displays or scatter plots, no substrate knowledge is required at all; instead, prebuilt Layers are available for public use, including a ScatterLayer, GraphLayer, MeshLayer, and many others, the most common of which are listed in Table 1.

In some cases, specific data requirements may mean that researchers cannot use these existing, prebuilt Layer implementations. If a developer decides to implement their own Layer from scratch, it can be trivially integrated into other visualizations, as all substrate Layers subscribe to the same simple interface and are interchangeable. For example, the big-data neuroscience community has developed a custom layer that visualizes larger-than-RAM imagery by shuttling data in and out of memory as it enters and leaves the substrate camera viewport. Social graph research teams have developed representations of graphs with enriched visual cues to signal node and edge attributes.

When research use-cases require customization, engineers can easily implement Layers that suit their specific need. Prebuilt Layers are sufficient for many applications, and require no engineering knowledge or ability from the end-user.

Our brownian-particle-motion example demonstrates how a developer can easily implement a custom Layer, while still taking advantage of prebuilt code. We envision that community users may request to merge commonly used Layers into the substrate codebase to extend native functionality and cover a diverse set of use-cases. As groups work together to achieve research goals, these researchers may separately develop Layers (e.g. a raw experimental Layer and an annotation Layer for the analysis) which can be combined in the same scene when needed.

### pytri

**Fig. 4:**
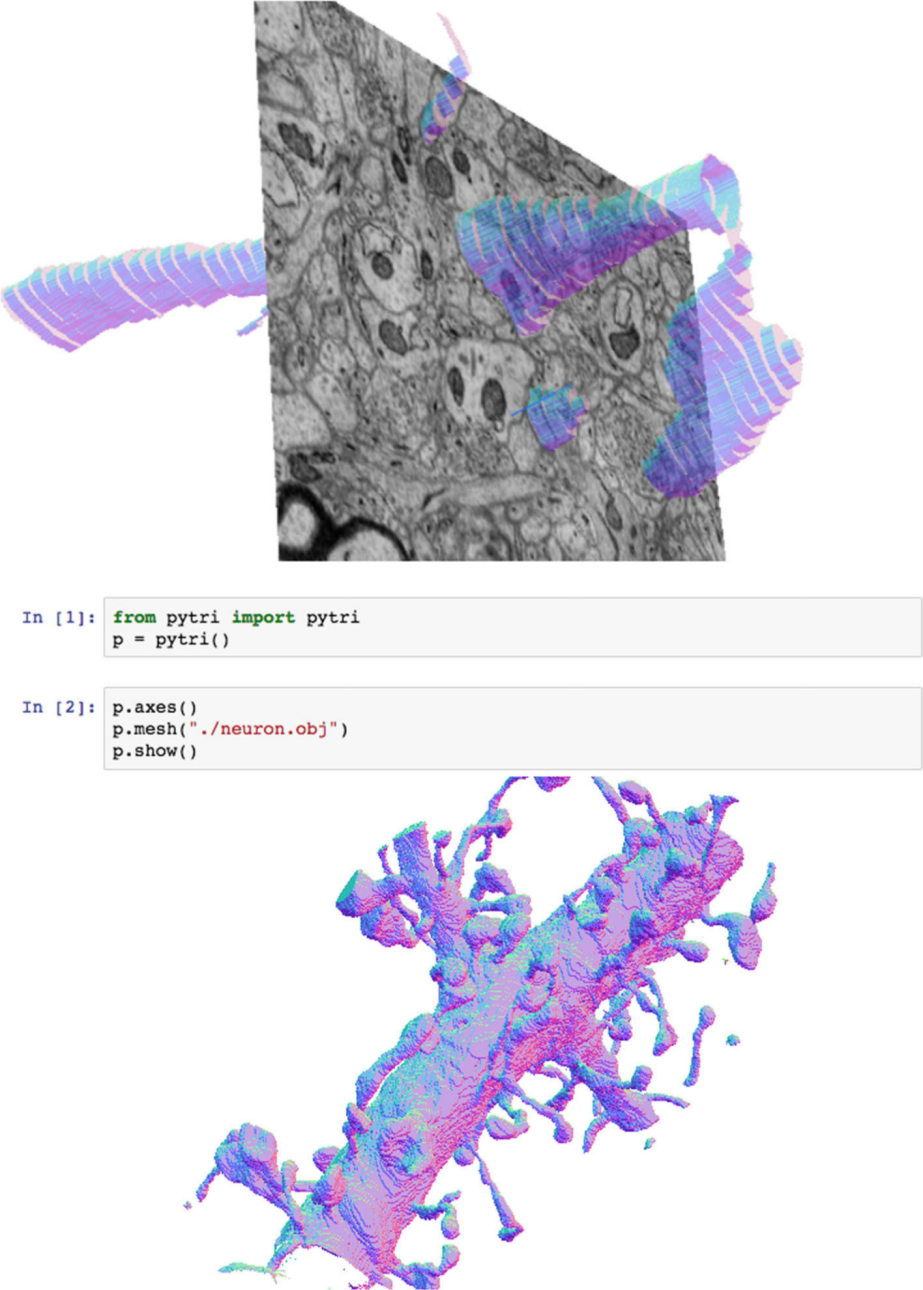
Here, pytri runs two simultaneous cells of different substrate visualizations inside a Jupyter notebook, using WebGL to enable real-time user interaction with the visualization. This high-detail mesh was generated using manual annotations from a recent electron microscopy study [19]

Requiring a scientist to exit their research environment in order to engage with visualizations reduces the time-efficacy of research [16, 17]. In order to provide a convenient, inline visualization solution for data scientists, we created pytri, a Python package that enables visualization of substrate Layers without leaving a Jupyter notebook [18] or other IPython environment (Fig. 4). Jupyter is a standard research platform for many communities. By bringing composable, extensible visualization to this platform, data scientists can quickly visualize and explore data in a familiar environment without needing to understand the underlying substrate codebase.

Our initial use-case demanded performant large-scale graph visualization. Though standalone visualization software existed for this scale of graph, our team found it onerous to exit our data science platform to view the raw data. Furthermore, we encountered issues with the performance of many popular 3D plotting libraries in Python, as they were unable to handle the size of graphs we needed to visualize. We built substrate to gracefully handle graph data containing many millions of edges. We then added corresponding hooks in pytri to empower users to create large-scale graph visualizations quickly without leaving the familiar Python environment.

pytri combines substrate capability with Python datatypes by leveraging the Jupyter Notebook IPython.display module, to which pytri delegates responsibility for interaction with the Jupyter DOM through both JavaScript (IPython.display.JavaScript) and HTML (IPython.display.HTML) [18].

These direct DOM manipulations, unlike the comparable Jupyter Widget architecture, enable pytri visualizations and data to persist even in a static HTML export of the Jupyter notebook. This enables the distribution of reproducible visualizations without requiring the end-user to install or configure software packages. In other words, researchers can produce static HTML files with interactive data visualizations which can be shared by email, by online publication, or through sharing the original source code.

## Results

The following use-cases illustrate substrate’s flexibility, idiomatic brevity, and ability to handle custom data. By lowering the overhead associated with task-switching between visualization and analysis, substrate provides an opportunity for a team to more intimately explore their data and iterate on analyses in realtime.

One of the key advantages of substrate is its use as a general framework for visualization across many domains. Here we highlight a few diverse applications that benefit from this work.

### Use case: analyzing biological imaging data

Biomedical research is one of many domains that benefits from the recent explosion in big data [20]. As such, there is pressing need for research tools that can adapt to the demands of large-scale datasets. Such a customizable and interactive framework for visualization can help researchers understand otherwise uninterpretable data. Volumetric imagery is one datatype that is particularly important both in biomedical research as well as in the clinical setting. Many existing frameworks for data visualization lack a lightweight tool that preserves the vital spatial relationships found in volumetric imagery.

When creating visualizations using substrate and pytri, the researcher has the speed and flexibility necessary to transform a typical visualization product from a static communication tool to a dynamic exploration aid. As an exercise in using pytri for data exploration and scientific communication, we present the following use-cases, showcasing similar opportunities across six orders of magnitude (e.g., magnetic resonance imaging (MRI) to electron microscopy (EM)). We focus on an exciting area of emerging research called connectomics, which focuses on estimating brain connectivity maps at various resolutions.

**Fig. 5:**
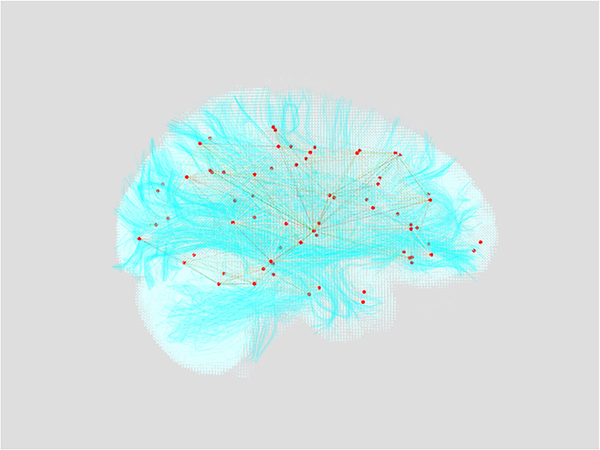
A view of MRI data using volumetric rendering. A graph of estimated brain region connectivity is rendered in red and yellow. Estimated fibers are rendered in cyan. The white brain volume is the direct output of a Diffusion Tensor Imagery MRI scan

We first investigate slices of MRI data volumes using ImageLayer, as demonstrated in Fig. 5. Navigation through the dense 3D data can be done programmatically through direct calls to the pytri API, or through the Jupyter UI, so the researcher never has to leave their data analysis environment. Beginning with this volumetric data representation, we can overlay fiber tracts representing estimates of major axonal bundles in the brain, and the derived nodes and edges associated with a connectome [21]. More specifically, the connectome is visualized using a GraphLayer overlaid on a MeshLayer of the surface of the patient’s brain as computed from structural MRI. When the analysis is ready to be shared, the researcher can easily package these visualizations for others by exporting the Jupyter notebook to an interactive HTML page.

With EM data, we show the flexibility of the tooling by displaying imagery slices, along with commonly used derived data representations such as meshes generated from manual or automated segmentation methods and skeleton traces. These tools are important to rapidly explore and validate large reconstructions. Because our visualization tools exist within an analytics environment, users can compute quantitative analyses in the same environment, reducing impediments to discovery. substrate’s layer-based framework allows the researcher to overlay multiple data sources, to reorient the view to focus on one detailed section of the data at a time, and to fully leverage data visualization in the research and sharing process.

### Use case: astronomical observational data

**Fig. 6:**
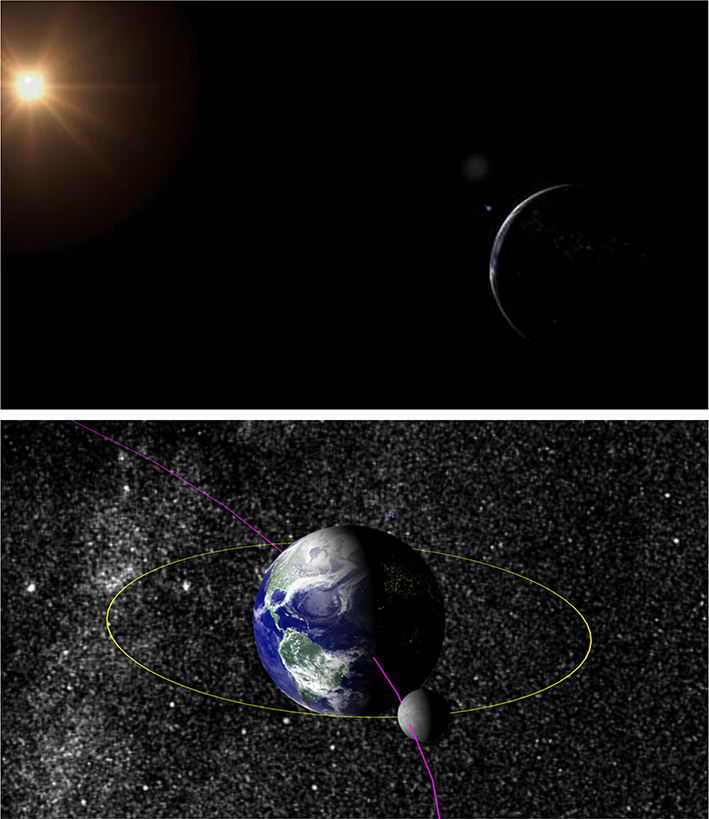
We use substrate to render the orbits of the Earth and Moon around the sun according to their realtime positions. A mesh representation of the International Space Station pulls live positional data from the internet [22, 23]. Orbital body sizes and trajectories are exaggerated for visibility

The civilian space community communicates mission details to a diverse audience, and visualization greatly enables public and collaborator understanding of mission planning and execution. The ability to quickly produce a visual summary of the mission can enable plan iteration and facilitate a discussion of alternative solutions. 3D visualization of a mission can also assist with exploring the complex maneuvers sometimes demanded in space exploration.

substrate is well-suited to display orbital and hyperbolic trajectories of bodies moving through outer space. In Fig. 6, we show the paths and bodies of the Earth/Moon system and the International Space Station (ISS) using only a FiberLayer to represent orbital paths and a MeshLayer (to render a downloaded 3D mesh of the ISS [22]) in substrate. The ISS position is updated in realtime to its current real-world position using the requestRender function of the Layer API (with data pulled from an online resource [23]). The sizes of the orbital bodies or satellites can be changed easily by removing, resizing, or reinserting the corresponding meshes. Many possible trajectories can be viewed in rapid succession by toggling their visibility in the scene. This use-case provides an example of how existing tools might be augmented through a simple, web-based visualization environment to engage the public and produce publication-ready graphics for community consumption.

### Use case 3: Geospatial information systems

Geospatial information is of interest to researchers in a variety of domains, including agriculture, architecture, and urban planning. Many of the existing state of the art geographic information systems (GIS) require standalone software installations, and visualizations are often handled in a separate application than that in which the initial data science is performed [24]. This requires researchers to switch between analysis and exploration, or else it constrains research pipelines to live inside of specialized visualization software such as QGIS [25] or SAGA [26].

**Fig. 7:**
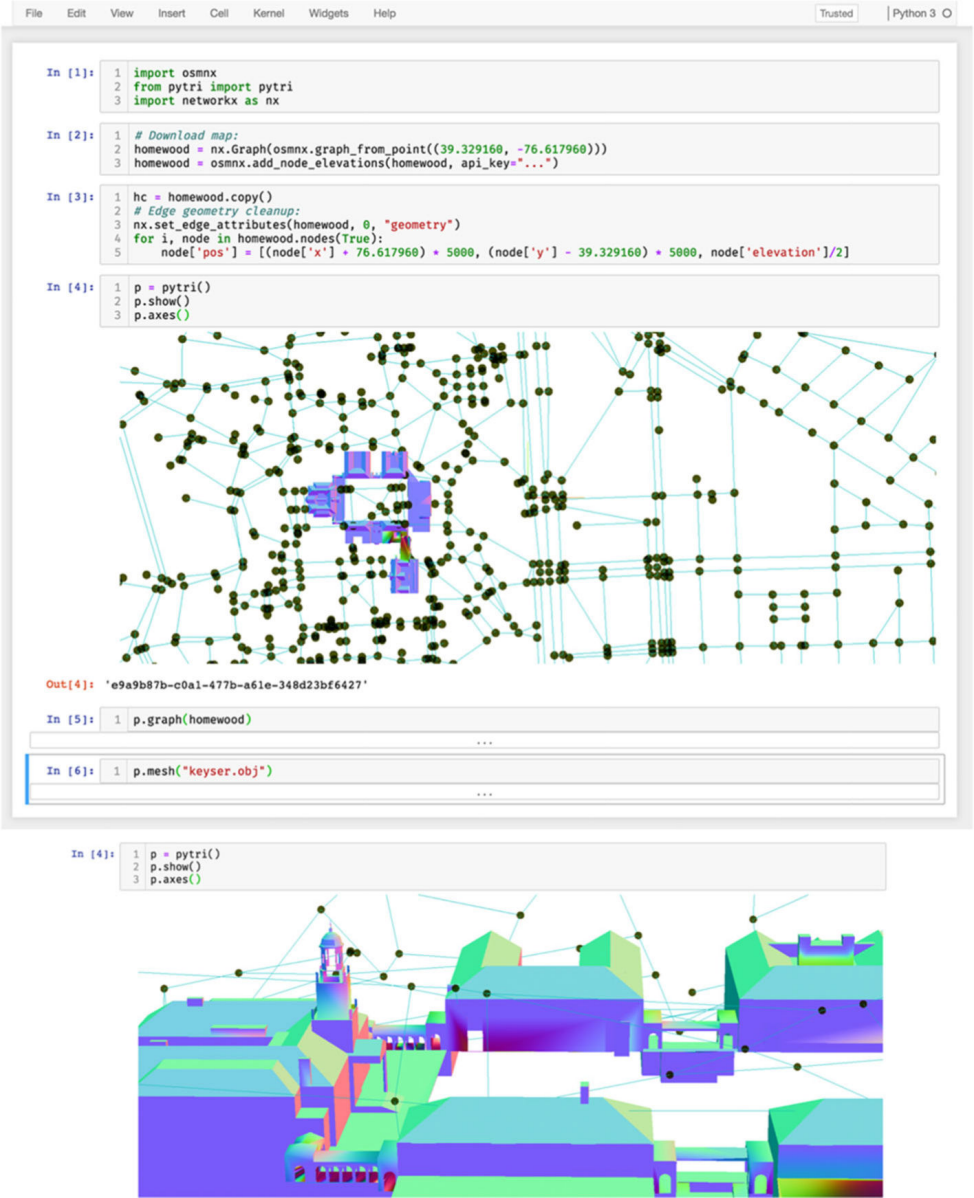
Using open street and path data from osmnx [24] and building meshes from *3D Warehouse* [27, 28], pytri displays a graph representation of the roads and paths surrounding the Johns Hopkins University Homewood campus. **Top**: The researcher renders an interactive 3D visualization of the region using pytri from within a code cell of a Jupyter notebook. **Bottom**: Clicking and dragging the visualization cell inside the notebook enables the user to navigate the visualization in 3D space. In this screenshot, the user renders a close-up angle of the Gilman Clock Tower mesh. Despite rendering large meshes and graphs side by side in 3D, this tool enables interactive, realtime, detailed visualization

Using pytri, GIS data can be visualized natively in Jupyter alongside corresponding analyses, and users may then visually explore the byproducts of this exploration without leaving the Jupyter notebook. Here, we perform a basic query of GIS data in the Johns Hopkins University Homewood Campus area using the osmnx Python package [24], one example of a tool one might use to download a large-scale graph. We then demonstrate the ability to coregister the visualizations of a graph of street connectivity alongside regions of interest and structure meshes downloaded from *3D Warehouse* [27, 28]. This provides a flexible framework to enrich a scene as additional sensors and data fusion products become available. We use pytri to visualize these data science products in a Jupyter notebook in Fig. 7.

This visualization uses a GraphLayer to represent streets, paths, and intersections as generated by the osmnx library in networkx.Graph format [14]. A MeshLayer is used to overlay a rendering of the Keyser Quad buildings in the same 3D coordinate frame.

Existing GIS visualization software tools often require that analysis is performed offline and data products are ingested — or else analyses are constrained to software-specific plugin architectures. The use of pytri enables simple 3D coregistered visualization of multimodal geographic data in a variety of data formats, including networkx graph and OBJ-formatted meshes.

## Discussion

We benchmarked substrate’s performance on several common Layer types in order to demonstrate the scalability and utility of this software for meso- to large-scale data. Performance was measured on consumer hardware (a 2017 MacBook Pro 15-inch model; 2.9 GHz Intel Core i7 processor and 16 GB 2133 MHz LPDDR3 Memory; Radeon Pro 560 4 GB, Intel HD Graphics 630, 1536 MB Graphics) and quantified using the Google Chrome built-in *Developer Tools* [29]. We report frames per second (FPS) where applicable, and we report the time from initial page-load to first-contentful-paint (FCP), a measure of how quickly the webpage can load and begin to render a data visualization [29]. FCP is a valuable metric of the realtime performance of the visualization system for an expected user workload. Because we were in some cases unable to generate a dataset large enough to meaningfully slow substrate rendering speeds, we have used FCP as a tool to quantify how these tools perform at scale.

**Fig. 8:**
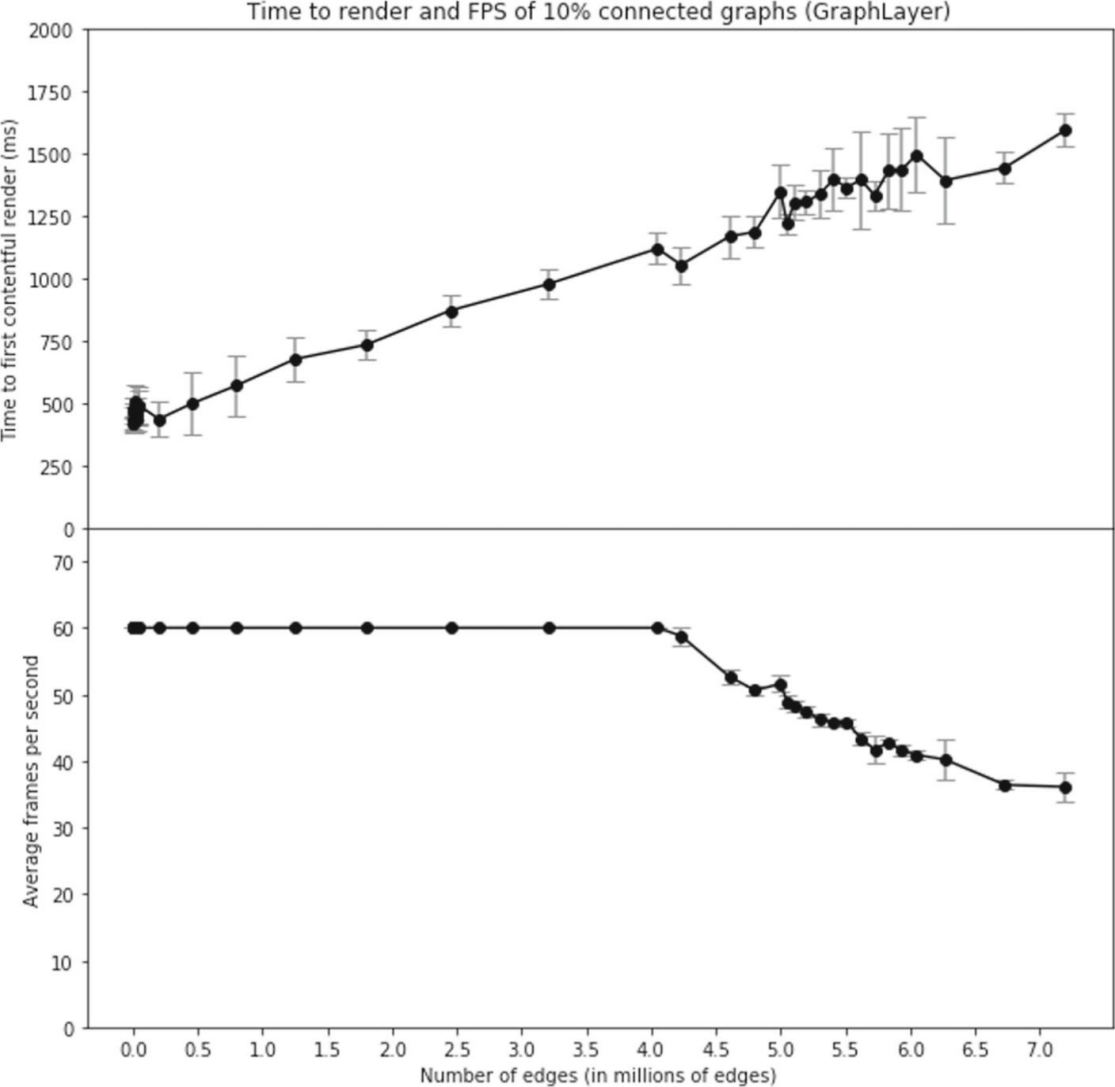
Time to First Contentful Paint, and frames per second (FPS) for large graphs, rendered using GraphLayer. 10 trials were performed for each size graph. Error bars represent standard deviation. Even very large graphs render faster than 2 s, and at greater frame-rate than 30 frames per second

We first tested a set of random, 10%-connected graphs, generated using the networkx.fast_gnp_random_graph function, and rendered using the substrate GraphLayer implementation. Frames-per-second (FPS) remained above 30 for all graphs measured in this test, as illustrated in Fig. 8.

The default RAM allocation of a modern browser tab (i.e., 1–3GB) constrains the ability to render larger graphs at interactive speeds on modern consumer hardware. Within these memory limitations, we showed that substrate was performant in an interactive environment. We estimate the largest fully connected graph that can fit in most modern browsers’ RAM to contain approximately four hundred million edges, when rendered using an adjacency-matrix style data structure as used in the substrate.GraphLayer above.

We then tested a set of icosphere OBJ-format meshes of varying subdivision level, generated and exported directly to OBJ format using the built-in functionality of Blender3D [30]. We selected this mesh shape as it maximized the proportion of the mesh visible to the rendering camera at all times during interactive zooming and camera movement for benchmarking worst-case performance. Renderer performance remained at an interactive speed (at or above 60FPS) for all mesh tests, and the time to first contentful paint remained low, as illustrated in Table 2. We advise that while users can load multiple gigabyte-size OBJ files into a substrate scene, researchers intending to manipulate larger meshes may find it advantageous to use a more memory-efficient mesh format.

These performance benchmarks are fully dependent upon the current Layer implementations, and future implementations, or more performant hardware, may improve performance and scalability further. If a research team needs to visualize raw mesh or graph data that are larger than the multi-gigabyte default RAM limitation, we recommend either manually increasing the memory limits of the browser, or developing a custom Layer implementation that selectively loads subsets of data from disk as they are needed.

## Conclusions

Similar to how web frameworks such as Angular [31], React [9], and Vue [10] popularized the *reusable component* model for maintainable web interface composition, our work emphasizes the reusability of *visualization* components by exposing an interface for discrete entities in a 3D scene. By combining several of these components, complex and deeply informative scenes can be designed with only minimal engineering effort. We share our implementation of this common interface in our software package substrate, which we provide as an open-source resource to the scientific community, and in pytri, a Python software package that enables the use of this common API without JavaScript knowledge. We intend that researchers unfamiliar with visualization technology will use pytri to compose visualizations in-line with their analyses, thus removing some of the activation energy required to produce and share reproducible, publication-ready graphics.

The list of components we provide is non-exhaustive, and we intend to support the community in efforts to increase the breadth of domains aided by substrate. Because many users may choose to conduct data science or research in languages besides Python, we intend to develop libraries for other common data science languages such as R and Julia, based upon ongoing community feedback.

Efforts are ongoing to natively support very large (out-of-RAM) dataset representations in both substrate and pytri libraries. Users with different tooling requirements, who require custom import formats, or who rely upon very large scale visualizations (e.g. graphs with billions of vertices and edges) may require additional engineering effort to fully leverage the substrate ecosystem. We also acknowledge the ongoing need for non-web-based visualization technologies when the RAM or performance limitations discussed above require that a user rely more heavily upon native tools. It is unlikely, for example, that the generalized tool substrate will replace professional and task-specific GIS tools such as QGIS, or professional 3D graphics tools such as Blender3D. Despite this, web-based — and in particular — Jupyter-based tools enable users to juxtapose their data science research with visualizations to not only improve a researcher’s ability to iterate on their hypotheses, but also to share their conclusions. Though web-based visualization may never fully replace more performant, local compute, it is our hope that tools such as substrate continue to enable more accessible and more shareable research.

As scientific datasets grow in size and complexity, communicating relevant data clearly and effectively is more important now than ever. Large-scale, multi-team efforts require portable and shareable visualizations that can be developed by several engineers simultaneously, and used by entire teams of both technical as well as non-technical individuals. It is our hope that tools like substrate and pytri will help support reproducible, reusable scientific discovery in the data science community. Our code and data are publicly available as described in the *Availability of Data and Material* section.

## Figures and Tables

**Table 1:** Available layers built into the substrate package

Layer name	Description	Useful for...
AxisLayer	Contains an RGB axis at the scene origin	...orienting the user in space
GraphLayer	A graph renderer with support for many millions of nodes or edges	...complex networks
ImageLayer	Import 2D images and render them as a plane	...displaying static iamgery
LightingLayer	Three-point lighting scheme, as commonly used in film and 3D graphics	...easy lighting setups
LineSegmentsLayer	A list of line segments to be rendered individually	...paths or wireframes
MeshLayer	Arbitrary 3D meshes, imported in common file formats such as OBJ	...arbitrary 3D solids
ScatterLayer	Point-clouds of many millions of points	...colored scatter plots
VolumeLayer	A visualization of dense 3D voxelwise data	...rendering volumetric data

**Table 2:** Performance statistics for rendering meshes of different sizes using substrate’s MeshLayer

File size (Megabytes)	Time to First Contentful Paint (milliseconds)	
	Mean	Std.

0.007	344.73	22.39
0.12	342.18	19.16
2.04	379.7	36.60
3.71	455.97	27.27
8.63	578.72	150.39
15.49	648.1	191.07

All meshes were read from the OBJ file-format on disk, and all examples below rendered at 60 frames per second on consumer hardware

## Abbreviations

API: Application programming interface
DOM: Document object model
EM: Electron microscopy
FCP: First contentful paint
FPS: Frames per second
GIS: Geospatial information systems
HTML: Hypertext markup language
ISS: International space station
MRI: Magnetic resonance imaging
RAM: Random access memory
